# Immobilization of *Aspergillus oryzae* IPT-301 Cells with High Transfructosylation Activity on Polyurethane Sponge for Fructooligosaccharide Production in Batch Mode

**DOI:** 10.1021/acsomega.6c07568

**Published:** 2026-10-14

**Authors:** Nathália Romani Barbosa, Ana Vieira, Fábio Aurélyo Silva Ferreira, Luara Cristina Alves, Sara Silverio, Sylma Carvalho Maestrelli, Sérgio Fernandes, Alfredo Eduardo Maiorano, Elda Sabino da Silva, Cristiane Ottoni, Sergio Villalba Morales, Rafael Firmani Perna, Michelle Xavier

**Affiliations:** † Graduate Program in Chemical Engineering, Institute of Science and Technology, 74347 Federal University of Alfenas, Poços de Caldas, Minas Gerais 37715-400, Brazil; ‡ Centre of Biological Engineering, University of Minho, Campus de Gualtar, Braga 4710-057, Portugal; § Graduate Program in Materials Science and Engineering, Institute of Science and Technology, 74347 Federal University of Alfenas, Poços de Caldas, Minas Gerais 37715-400, Brazil; ∥ Bionanomanufacturing Center, 154620 Institute for Technological Research, São Paulo, São Paulo 05508-901, Brazil; ⊥ Biosciences Institute, São Paulo State University, São Vicente, São Paulo 11330-900, Brazil; # Graduate Program in Food Science and Technology, Federal University of Tocantins, Palmas, Tocantins 77001-090, Brazil

## Abstract

A stable and active biocatalyst for fructooligosaccharide (FOS) production was synthesized by immobilizing *A. oryzae* IPT-301 cells on a polyurethane sponge (PS) during submerged cultivation. Comparisons between immobilized and cross-linked immobilized cells revealed similar biochemical properties. The highest cell concentration immobilized in PS (11.89 ± 2.29 g cells g support^–1^) was obtained after 32 h. Both biocatalysts performed optimally at 50 °C, pH 5.5, and 480.2 g L^–1^ sucrose, showing a *V*_max_ of 3500 μmol g^–1^ min^–1^ and high selectivity for 1-kestose (28.23%). They exhibited a half-life of 300 min at 50 °C, retained 40% activity after 28 days of storage and 50% activity after 12 reaction cycles. FOS productivity reached 116 g L^–1^ h^–1^ with a 24% yield. Statistical analysis confirmed that cross-linking did not significantly influence performance. The results show that *in situ* immobilization on PS is a simple, efficient strategy for producing stable biocatalysts for FOS synthesis.

## Graphical abstract

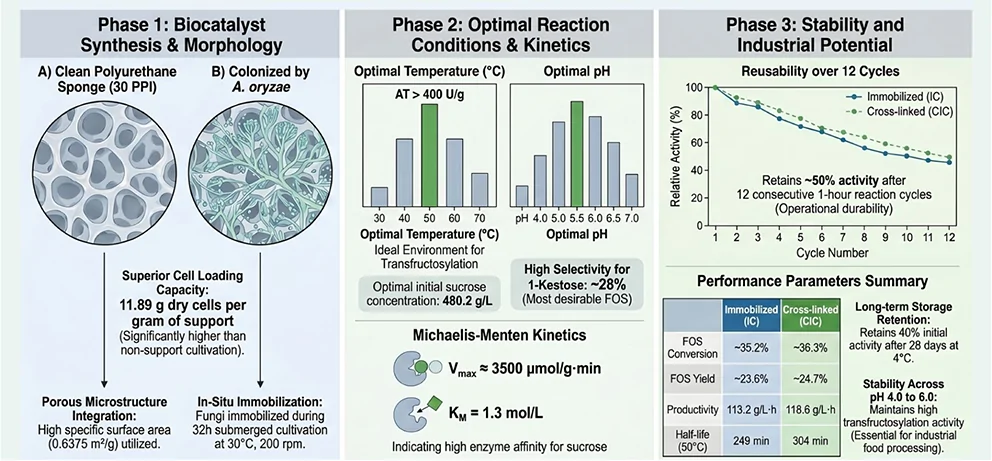

## Introduction

Fructooligosaccharides (FOS) are important low-calorie sugars and prebiotics widely used in the food industry to prepare functional foods due to their beneficial effects on human and animal health.^1–3^ Several studies have emphasized that FOS consumption enhances calcium and magnesium absorption, promotes bacterial selectivity in the intestinal microbiota, and helps reduce serum cholesterol levels. Additionally, they contribute to the treatment of conditions such as anemia, hypertension, and renal failure.^2–4^

These sugars are fructose oligomers produced by the transfructosylation of sucrose molecules, a reaction catalyzed by microbial fructosyltransferase enzymes (FTase; E.C.2.4.1.9).^5–7^ These enzymes are mainly produced by fungi from the genera *Aureobasidium*, *Penicillium*, and *Aspergillus*.^8–10^ Principally, cells from the strain *Aspergillus oryzae* IPT-301 have exhibited higher transfructosylation activity than those of several other species and have been applied as biocatalysts for FOS production in batch and continuous reactors at the laboratory scale.^10–12^ However, these biocatalysts can deactivate rapidly and are not easily reusable.

Immobilization of cells is a promising strategy for producing biocatalysts with high stability.^13^ Immobilized cells have shown advantages such as enhanced maintenance of biological functions, high resistance to adverse conditions in the reaction media, and increased productivity.^13,14^ The immobilization process also facilitates the reuse and separation of the biocatalyst from the reaction medium, as well as its application in packed-bed reactors.^13,14^ Studies on cell immobilization for FOS production have reported the use of different immobilization techniques and supports, including gluten, calcium or sodium alginate for cell entrapment;^15–17^ or immobilization during cell cultivation on agro-industrial byproducts (e.g., walnut shell, almond shell, chestnut shell), synthetic products (e.g., polyurethane sponge, stainless steel sponge, glass sponge, fiberglass wool) or natural inorganic materials (e.g., pumice stone, zeolite molecular sieves).^8,18^

Currently, the cells of *A. oryzae* IPT-301, which have great potential for FOS production, have only been immobilized by entrapment in calcium alginate spheres^19^ and by cross-linking with glutaraldehyde.^20^ Although both techniques enhanced the operational stability of the catalytic cells, they also required the use of chemical agents and a long synthesis time. Also, cell entrapment in the gel can impose mass-transport limitations on the substrate to the active site. Cell immobilization on a porous support is an effective strategy for improving the stability and enzymatic activity of biocatalysts. In this context, and unlike previously reported methods for *A. oryzae* IPT-301, the novelty of this study lies in the *in situ* immobilization onto a macroporous polyurethane sponge (PS) during submerged cultivation. This strategy eliminates the need for complex presynthesized matrices and cumbersome multiphase chemical protocols. By directly colonizing the open-pore structure during growth, high cell loading and catalytic activity are achieved in a shorter cultivation time, avoiding mass-transfer limitations while providing enhanced operational stability for FOS synthesis.^8,18^ Therefore, the main goal of this work was to synthesize stable, highly active biocatalysts for FOS production by immobilizing *A. oryzae* IPT-301 cells onto a macroporous PS and subsequently cross-linking them. The biochemical properties and stability of these biocatalysts were then evaluated during the biocatalytic process.

## Materials and Methods

### Materials

The fungal strain *A. oryzae* IPT-301 was provided by the Industrial Biotechnology Laboratory of the Technological Research Institute of São Paulo (LBI/IPT, São Paulo, Brazil). Commercial polyurethane sponges with 30 pores per inch were purchased at a local grocery store (Poços de Caldas, Brazil). Sucrose, yeast extract, monopotassium phosphate (KH_2_PO_4_), manganese­(II) chloride tetrahydrate (MnCl_2_·4H_2_O), and iron­(II) sulfate heptahydrate (FeSO_4_·7H_2_O) were purchased from Labsynth (Diadema, Brazil). Sodium nitrate (NaNO_3_), magnesium sulfate heptahydrate (MgSO_4_·7H_2_O), sodium hydroxide (NaOH), 3,5-dinitrosalicylic acid (C_7_H_4_N_2_O_7_), Glutaraldehyde solution grade I (25% in water), sodium borohydride (NaBH_4_), potassium sodium tartrate (KNaC_4_H_4_O_6_·4H_2_O), and tris­(hydroxymethyl)­aminomethane (NH_2_C­(CH_2_OH)_3_) were purchased from Dinamica (Diadema, Brazil). The enzymatic colorimetric kit GOD-PAP for glucose determination was supplied by LaborLab (Guarulhos, Brazil). Fructooligosaccharide standards were purchased from Wako Chemicals (Richmond, USA). All reagents used were of analytical grade.

## Methods

### Characterization of the Immobilization Support

The morphology of the polyurethane sponge (PS) was observed by scanning electron microscopy (SEM) using a Zeiss EVO MA-10 equipment, operating with an acceleration voltage of 20 kV, a spot size of 4.8, and a working distance of 9.6 mm. The sample was previously dried at 60 °C for 2 h in a Micromeritics VacPrep 61 Sample Degas System; the B.E.T. specific surface area of the PS was determined at 77 K by nitrogen physisorption using a Micromeritics Gemini VII analyzer; the apparent density of the sample was determined according to ASTM D3574.

### Production and Immobilization of Biocatalytic Cells

Biocatalytic cells from *A. oryzae* IPT-301 were produced by submerged cultivation in 50 mL of synthetic culture medium, pH 5.5, with the following composition (in % w v^–1^): sucrose 15.0, yeast extract 0.5, NaNO_3_ 0.5, KH_2_PO_4_ 0.2, MgSO_4_·7H_2_O 0.05, MnCl_2_.4H_2_O 0.03 and FeSO_4_·7H_2_O 0.001.^5^ About 20 macroporous PS (cubes with 0.8 cm edges) were added to the culture medium to immobilize the microbial cells. All cultivation media were sterilized at 120 °C for 20 min (Vertical Autoclave Phoenix Luferco, Araraquara, Brazil). The flasks were inoculated with 500 μL of spore suspension (1 × 10^7^ spores mL^–1^) and incubated in a rotary shaker (Tecnal TE-4200, Piracicaba, Brazil) at 30 °C and 200 rpm for 32 h.^12,20,21^ After cultivation, the culture broth was vacuum-filtered to retain the immobilized cells (IC) with transfructosylation activity. The morphology of the biocatalytic cells immobilized on the PS was analyzed by SEM under the same conditions as previously specified to determine the morphology of the PS before cell cultivation.

### Cross-Linking Assays of Immobilized Biocatalytic Cells

The cross-linking assays using the immobilized biocatalytic cells were performed based on an adaptation of the methods reported by Garcia et al.^20^ and Ribeiro et al.^22^ About 2.0 g of wet mass of IC (equivalent to nine cubic polyurethane particles) were added to an Erlenmeyer flask, followed by 16.8 mL of a glutaraldehyde solution (25 wt % in water) previously dissolved in 183.2 mL of 0.2 mol L^–1^ tris-acetate buffer, pH 7.9. The reaction was performed at 25 °C and 200 rpm for 45 min on a Dubnoff water bath (Novatecnica, NT230, Piracicaba, Brazil) and interrupted for a further 30 min by the addition of 2 mL of sodium borohydride P.A. (NaBH_4_) at 100 g L^–1^, previously dissolved in a sodium hydroxide P.A. solution (1 × 10^–3^ mol L^–1^). The cross-linked immobilized cells (CIC) were vacuum-filtered, washed with distilled water, and stored under refrigeration at 4 °C in 0.2 mol L^–1^ Tris-acetate buffer (pH 5.5) for subsequent biochemical characterization.

### Assays for the Characterization of Biochemical Properties

#### Influence of Temperature and pH on Enzymatic Activity

The enzymatic activity of the IC and CIC was measured within the temperature range (40 °C ≤ T ≤ 60 °C, with ΔT = 5 °C) at pH 5.5, and pH range (4.0 ≤ pH ≤ 7.0, with ΔpH = 0.5), at 50 °C, using a sucrose P.A. solution (48% w v^–1^) as substrate. The transfructosylation activity was measured under standard conditions.

#### Influence of Substrate Concentration on the Enzymatic Activity and Estimation of the Kinetic Parameters

The transfructosylation activities of both biocatalysts (IC and CIC) were determined at 50 °C in a reaction medium composed of 1.2 mL of Tris-acetate buffer solution (0.2 mol L^–1^, pH 5.5) and 3.7 mL of sucrose P.A. solution (75.5, 151.0, 302.0, 377.5, 480.2, and 604.0 g L^–1^). Approximately 0.7 g of wet cell biomass (equivalent to three cubic PS) of both IC and CIC biocatalysts was added to the reaction medium. The sucrose, FOS, glucose, and fructose concentrations were determined by high-performance liquid chromatography (HPLC), as described by Maiorano et al.^10^

The reaction rate of sucrose and products (*μ*mol g^–1^ min^–1^) were determined through a differential mole balance in a batch reactor (eq 1), as described by Fogler,^23^ where *V* is the reaction volume (L), *MM*_*i*_ is the molar mass of the molecule i (g mol^–1^), *W* is the mass of biocatalyst (g), *t* is the reaction time (min), and *C*_*i*_ and *C*_*io*_ are the concentrations of the molecule i at the end and at the beginning of the reaction (*μ*g L^–1^), respectively.

1 $$r_{i}=\frac{V}{MM_{i}\times W}\frac{\left(C_{i}-C_{iO}\right)}{t}$$

The kinetic parameters were estimated through nonlinear fitting of the Michaelis–Menten model (eq 1) and a zero-order kinetic model (eq 2) to the experimental data.^5,24^

2 $$r_{i}=\frac{V_{max}\left[S\right]}{\left[S\right]+K_{m}}$$

3 $$r_{i}=k_{i}$$

Where r_i_ is the reaction rate for molecule i (*μ*mol g^–1^ min^–1^), *V*_max_ is the maximum reaction rate achieved by the system, at saturating substrate concentration (*μ*mol g^–1^ min^–1^), [S] is sucrose concentration (mol L^–1^), K_M_ is the Michaelis–Menten constant (mol L^–1^) and *k* is the zero-order reaction constant for molecule i­(*μ*mol g^–1^ min^–1^).^23,25^

The reaction selectivity for product (*S*_*i*_) was obtained using eq 3, in which its reaction rate (*r*_*i*_) was divided by the sum of the reaction rates of all products (*r*_products_). The index *i* represents the evaluated reaction product, including glucose (G), fructose (F), 1-kestose (GF_2_), nystose (GF_3_), and 1-β-fructofuranosylnystose (GF_4_). Sucrose conversion was calculated as the ratio of the substrate concentration consumed during the reaction and its initial concentration (eq 4), as described by Fogler.^23^

4 $$S_{i}=\frac{r_{i}}{r_{produtos}}=\frac{r_{i}}{r_{G}+r_{F}+r_{GF_{2}}+r_{GF_{3}}+r_{GF_{4}}}$$

#### Operational Stability

Approximately 0.7 g of wet cell biomass (equivalent to three cubic PS) of both IC and CIC biocatalysts was added to the reaction medium containing 3.7 mL of sucrose P.A. solution (48% w v^–1^) and 1.2 mL of Tris-acetate buffer solution (0.2 mol L^–1^, pH 5.5). The enzymatic reactions were conducted under standard experimental conditions. At the end of each batch cycle, corresponding to 1 h of reaction, the biocatalyst was removed from the reaction medium by vacuum filtration and subsequently reintroduced into a fresh reaction medium under identical conditions. Transfructosylation activities and FOS concentration were determined as functions of the number of consecutive reaction cycles. The performance parameters, conversion (X), yield (Y), and productivity (P), were calculated according to eqs 5–7, respectively.^23,26^

5 $$X\left(\%\right)=\left[\frac{C_{inicial}-C_{final}}{C_{Inicial}}\right]\times 100$$

6 $$Y\left(\%\right)=\left[\frac{C_{FOS}}{C_{inicial}}\right]\times 100$$

7 $$P=\frac{C_{FOS}}{t}$$

Where *C_initial_* and *C_final_* are the initial and final concentrations of the substrate (sucrose) in the reaction medium (in g L^–1^), respectively; *C_FOS_* is the concentration of FOS produced by the enzymatic reaction (in g L^–1^), and *t_reaction_* is the reaction time (in h).

#### Thermal Stability Assays and Estimation of the Kinetic and Thermodynamic Parameters

Approximately 0.7 g of wet cell biomass (equivalent to three cubic PS) of both IC and CIC biocatalysts was incubated in Tris–acetate buffer (0.2 mol L^–1^, pH 5.5), in the absence of substrate, within a wide temperature range (30 °C, 40 °C, 50 °C, and 60 °C). Samples were collected at predefined time intervals (1, 2, 4, 8, 16, and 36 h) and immediately cooled in an ice–water bath for 5 min. The remaining activity was determined under standard conditions. The first-order thermal deactivation constant (k_D_, in min^–1^) was estimated by fitting the Sadana and Henley model^27^ to the experimental data on residual activity versus incubation time.^19,21,28^ The half-life (*t*_1/2_, in min), enthalpy of activation of denaturation (ΔH_D_, in kJ mol^–1^), Gibbs energy of activation of denaturation (ΔG_D_, in kJ mol^–1^), and entropy of activation of denaturation (ΔS_D_, in kJ mol^–1^ K^–1^) were determined according to Faria et al.^24^ and Gonçalves et al.^19^

#### pH and Storage Stability Assays

For the pH stability assays, approximately 0.7 g of wet cell biomass (equivalent to three cubic PS) of both IC and CIC biocatalysts was incubated in a Tris-acetate buffer solution (0.2 mol L^–1^) over a pH range of 3.5–8.5 (ΔpH = 0.5), in the absence of substrate, and maintained at 4 °C for 24 h. For the storage stability assays, IC and CIC biocatalysts were kept in a Tris-acetate buffer (0.2 mol L^–1^, pH 5.5) without substrate at 4 °C and sampled after 1 (24 h), 2, 5, 7, 9, 14, 21, and 28 days. In both assays, transfructosylation activity was determined under standard reaction conditions.

### Analytical Methods

#### Enzymatic Activity Assays

The transfructosylation activity was determined according to Gonçalves et al.,^19^ Garcia et al.,^20^ and Araújo et al.^21^ Approximately 0.7 g of wet cell biomass (equivalent to three cubic PS) of both IC and CIC biocatalysts was incubated with 3.7 mL of sucrose solution (48% w v^–1^) and 1.2 mL of Tris-acetate buffer (0.2 mol L^–1^, pH 5.5). The enzymatic activity assays were performed at 50 °C and 190 rpm for 60 min, and stopped by boiling water for 10 min and an ice bath for 5 min. The unit of enzymatic activity was defined as the amount of enzyme that transfers 1 μmol of fructose per minute per gram of dry cells under the chosen experimental conditions. The concentrations of reducing sugars and glucose were quantified using the 3,5-dinitrosalicylic acid (DNS) method and the enzymatic Glucose kit (GOD-PAP), respectively.^12,22,24^

#### Chromatographic Analysis

Sucrose, glucose, fructose, and FOS concentrations in the samples were analyzed by high-performance liquid chromatography (HPLC) with a Shodex SC 1011 (300 × 7.8 mm, Waters, USA) column, and a system composed of a W515 isocratic pump, a refractive index detector W2414, and a data processor with register (Waters, USA). The samples were eluted with an EDTA-Ca­(Na)_2_ solution at 5 × 10^–2^ mol L^–1^, at a flow rate of 0.4 mL min^–1^. The temperature of the column was maintained at 72 °C. FOS concentration was considered as the sum of 1-kestose (GF_2_), nystose (GF_3_) and 1-β-fructofuranosylnystose (GF_4_) concentrations.^10^

### Statistical Analysis

Experiments subjected to statistical analysis were performed in triplicate, and the results are expressed as the mean ± standard deviation (SD), where applicable. Statistical comparisons were performed by analysis of variance (ANOVA), followed by Tukey’s honest significant difference (HSD) test at a 95% confidence level (*p* < 0.05), where applicable. Different letters indicate statistically significant differences among the evaluated conditions, whereas identical letters indicate no statistically significant difference (*p* ≥ 0.05). When IC and CIC were compared at the same experimental condition, ns indicates no statistically significant difference, whereas * indicates a statistically significant difference (*p* < 0.05). Error bars in the figures represent the standard deviation of three independent measurements (*n* = 3). For parameters derived from measurements without independent experimental replicates, the results are presented descriptively and were not subjected to inferential statistical analysis.

## Results and Discussion

### Characterization of the Polyurethane Sponge before and after the Immobilization of Cells

Figure 1A shows the PS before the immobilization of the *A. oryzae* IPT-301 cells, where the pores and grooves of the sponge structure are clearly visible and heterogeneous. The heterogeneous formation of pores during PS manufacturing results from the reaction between polyol and isocyanate, which releases gases (typically CO_2_).^29–31^ The reaction rate and the uniformity of reagent mixing directly influence pore homogeneity. The PS has a low apparent density (19.72 ± 0.56 kg m^–3^), which is consistent with the literature^32,33^ and with SEM images, in which interconnected pores, open cells, and high porosity were observed. The specific surface area obtained by the B.E.T. technique was 0.6375 m^2^ g^–1^, similar to results reported in the literature.^34^ Free *A. oryzae* IPT-301 cells generally grow as small pellets of homogeneous mycelium formed by nonseptate hyphae and lacking fruiting bodies or spores, features that are characteristic of the mycelial growth of a filamentous fungus.^12,22,35^ However, as a filamentous fungus, it can readily adapt to porous materials. In this study, SEM analysis was primarily used to verify the immobilization and colonization of *A. oryzae* IPT-301 cells on the PS by comparing the support before and after cell cultivation. Figure 1B shows cell growth along the grooves and filaments of the PS after 32 h of cultivation.

**Figure 1:**
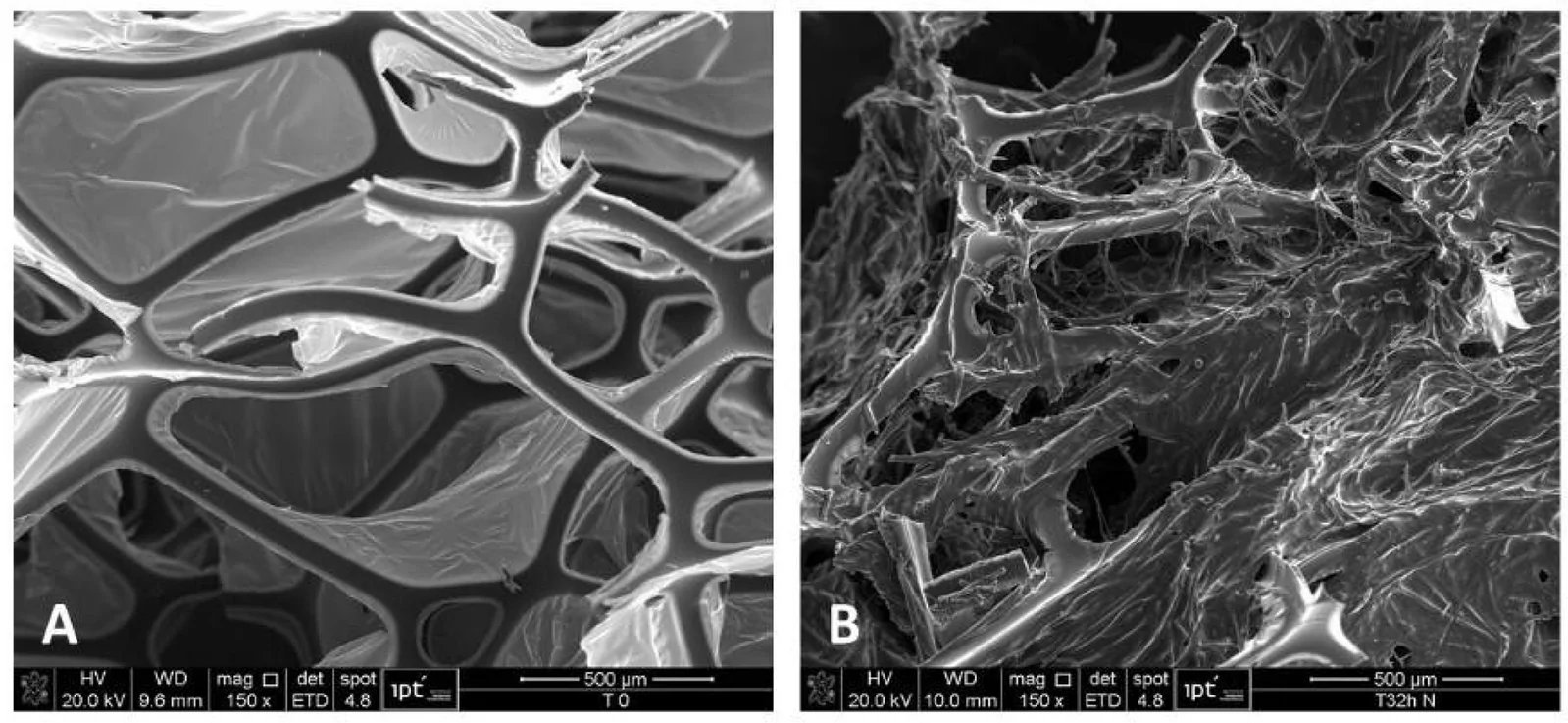
Scanning electron microscopy (SEM) images of PS: (A) before cell cultivation and (B) after immobilization of *A. oryzae* IPT-301 cells. Cultivation time: 32 h; magnification: 150×; scale bars: 500 μm.

Figure 2 shows cell growth on the PS and its transfructosylation activity as a function of the cultivation time. The highest concentration of IC (11.89 ± 2.29 g dry cells g support^–1^) was reached at 32 h, favored by vigorous agitation of the culture medium (200 rpm), which enhanced oxygen and nutrient transfer, promoting both growth and immobilization of the fungal cells.^36^ From this point onward, a reduction in microbial growth was observed, possibly associated with the depletion of essential nutrients for cellular metabolism, such as carbon and nitrogen sources in the culture medium, and/or the accumulation of metabolic products toxic to microbial cells.^37^ The concentration of IC obtained in this work was considerably higher than that reported by Mussatto et al.,^18^ who obtained a concentration of 0.48 g cells g support^–1^, after 48 h of cultivation, at 28 °C and 160 rpm, using a synthetic culture medium, the fungus *Aspergillus japonicus*, and 0.3 cm^3^ of sponges. It is also noteworthy that the higher cell concentration obtained in this work was achieved in a considerably shorter cultivation time than those reported by Cunha et al.^5^ and Ottoni et al.^38^ These authors reported the production of 9.35 ± 1.26 g L^–1^ and 4.85 g L^–1^ of *A. oryzae* IPT-301 cells, respectively, after 48 h of cultivation under the same experimental conditions used in this work, but without the addition of a support material. These results indicate that cultivating *A. oryzae* IPT-301 PS increased the cell growth rate. The high concentration of IC is mainly attributed to the size and distribution of the pores in the support, which favor the diffusion of gases and substrate.^39^

**Figure 2:**
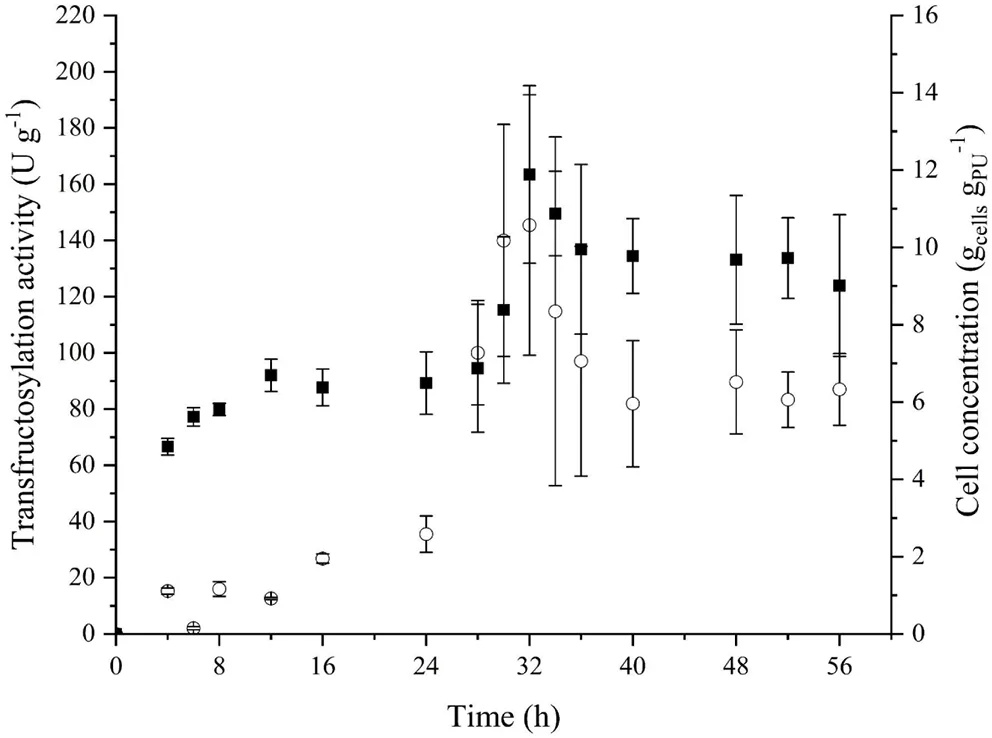
*A. oryzae* IPT-301 cell growth on cubic PS matrices (0.8 × 0.8 × 0.8 cm) as a function of culture time. (■) Cell concentration and (○) transfructosylation activity. Transfructosylation activity was determined in a reaction medium consisting of a 48% (w v^–1^) sucrose solution in 0.2 mol L^–1^ Tris-acetate buffer (pH 5.5), at 190 rpm and 50 °C for 60 min. Data are expressed as mean ± SD (*n* = 3). Error bars represent the standard deviation.

Furthermore, the maximum transfructosylation activity of the IC (145.43 ± 46.30 U g^–1^) was also obtained at 32 h of cultivation, indicating that this cultivation time allowed the formation of a biocatalyst with both high cell concentration and catalytic activity, which are important characteristics for efficient enzymatic FOS production. Finally, the cultivation medium became slightly more acidic over time, with the pH decreasing from 5.5 to 4.4. This likely occurred because microorganisms such as *A. oryzae* produce acid proteases that cleave peptide bonds, releasing amino acids and thereby acidifying the culture medium.^40,41^

### Influence of Reaction Temperature and pH on Enzymatic Activity

The effect of temperature on the transfructosylation activity of *A. oryzae* IPT-301 cells (IC and CIC) is shown in Figure 3. The highest enzymatic activities (278.85 ± 52.27 U g^–1^ and 383.09 ± 43.14 U g^–1^) were observed at 50 °C for IC and CIC, respectively. One of the main characteristics of filamentous fungi is their ability to grow on solid surfaces. Thus, the immobilization of fungal hyphae on the sponge may enhance resistance to thermal denaturation.^42^ Moreover, the lower viscosity of the reaction medium at 50 °C facilitates diffusion of both substrates and reaction products, likely contributing to the increase in enzymatic activity. Conversely, at higher temperatures, enzyme denaturation may occur, leading to a decline in catalytic activity. These findings are consistent with previously reported data for *A. oryzae* IPT-301. When free cells were used, the optimal temperature was above 46 °C.^5^ In contrast, glutaraldehyde-cross-linking cells exhibited maximum transfructosylation activity between 60 and 65 °C,^20^ while glutaraldehyde-cross-linking cells encapsulated in calcium alginate showed the highest activity in the range of 40–60 °C.^19^

**Figure 3:**
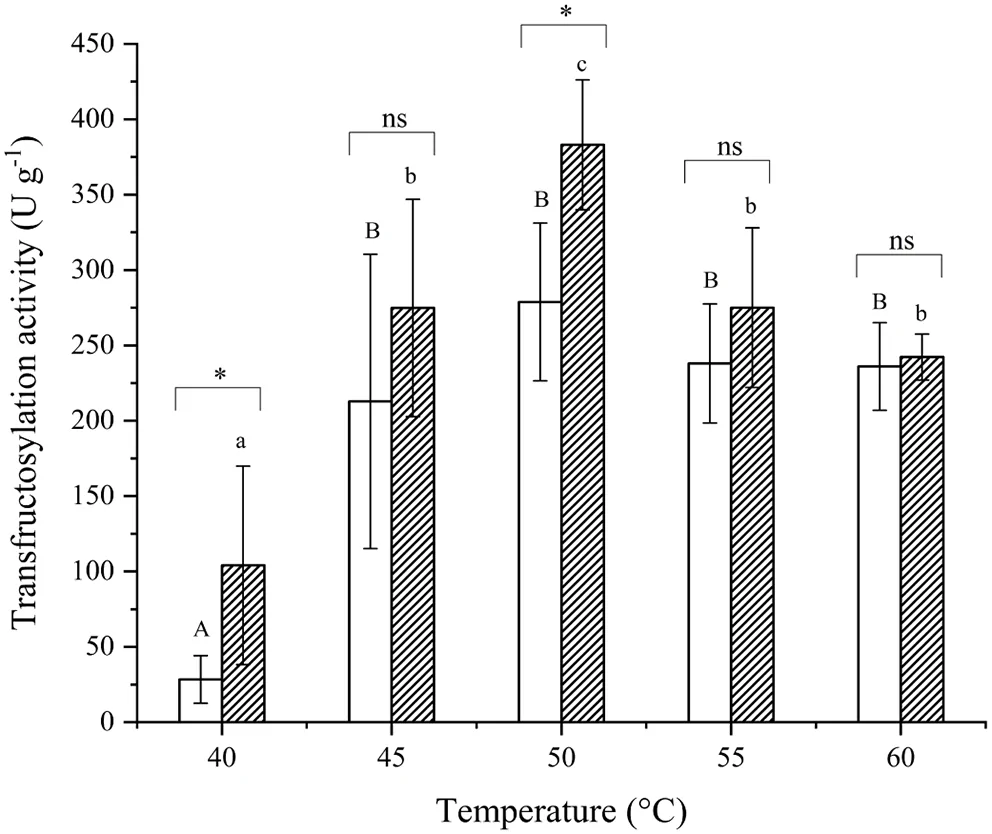
Influence of reaction temperature on the transfructosylation activities of *A. oryzae* IPT-301 IC and CIC on cubic PS matrices (0.8 × 0.8 × 0.8 cm). Reaction conditions: 48% (w v^–1^) sucrose solution in 0.2 mol L^–1^ Tris-acetate buffer (pH 5.5), 190 rpm, and 60 min. IC (open bars) and CIC (hatched bars). Data are expressed as mean ± SD (*n* = 3). Error bars represent the standard deviation. Different uppercase and lowercase letters indicate statistically significant differences among temperatures for IC and CIC, respectively, according to Tukey’s HSD test (*p* < 0.05). For comparisons between IC and CIC at the same temperature, ns indicates no statistically significant difference, whereas * indicates a statistically significant difference (*p* < 0.05).

The highest enzymatic activity values (328.49 ± 77.34 and 420.87 ± 94.76 U g^–1^) were observed at pH 5.5 for IC and CIC, respectively (Figure 4). Although CIC showed numerically higher mean activity than IC over the pH range of 4.0–6.5, no statistically significant differences were observed between the two biocatalysts at the same pH (*p* > 0.05). These findings are consistent with previous studies reporting pH 5.5 as a favorable condition for the transfructosylation activity of *A. oryzae* IPT-301 whole cells,^5^ glutaraldehyde-cross-linked cells,^20^ and glutaraldehyde-cross-linked cells encapsulated in calcium alginate.^19^ In general, cross-linking has been reported to mitigate the loss of enzymatic activity, as enzymes associated with the cell surface may become more resistant to variations in the ionic strength of the reaction medium.^20,37,41^ This stabilization effect may explain the improved transfructosylation activity reported for cross-linked cells in previous studies. However, under the conditions evaluated in the present study, the additional cross-linking step did not result in a statistically significant increase in activity compared with IC.

**Figure 4:**
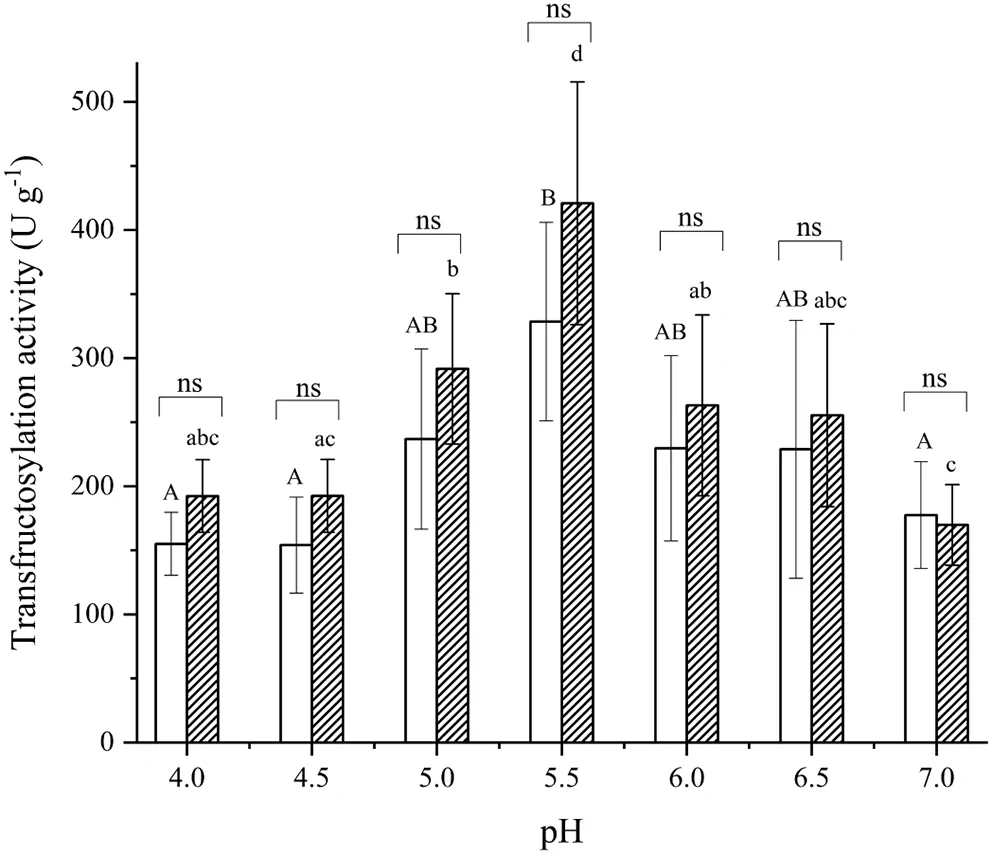
Influence of the pH of the reaction medium on the transfructosylation activities of *A. oryzae* IPT-301 IC and CIC in cubic PS matrices (0.8 × 0.8 × 0.8 cm). IC (open bars) and CIC (hatched bars). Reaction conditions: 48% (w v^–1^) sucrose solution, 0.2 mol L^–1^ Tris-acetate buffer, 190 rpm, 50 °C, and 60 min. Data are expressed as mean ± SD (*n* = 3). Error bars represent the standard deviation. Different uppercase and lowercase letters indicate statistically significant differences in pH for IC and CIC, respectively, according to Tukey’s HSD test (*p* < 0.05). For comparisons between IC and CIC at the same pH, ns indicates no statistically significant difference, whereas * indicates a statistically significant difference (*p* < 0.05).

### Effect of Substrate Concentration on Enzymatic Activity and Kinetic Parameters

Figure 5 illustrates the effect of sucrose concentration on the transfructosylation activity of *A. oryzae* IPT-301 IC and CIC on the PS matrix. It was observed for both biocatalysts that the highest enzymatic activities, exceeding 650 U g^–1^, were achieved at a sucrose concentration of 480.2 g L^–1^. Within the substrate-concentration assay, the increase in transfructosylation activity with increasing sucrose concentration can be attributed to greater substrate availability at the catalytic sites and increased formation of the fructosyl-enzyme intermediate.^9^ At high sucrose concentrations, sucrose also acts as an efficient fructosyl acceptor, favoring transfructosylation and FOS formation.^5^ The progressive increase followed by a tendency toward saturation is consistent with the Michaelis–Menten-type behavior observed for the biocatalysts.^25^

**Figure 5:**
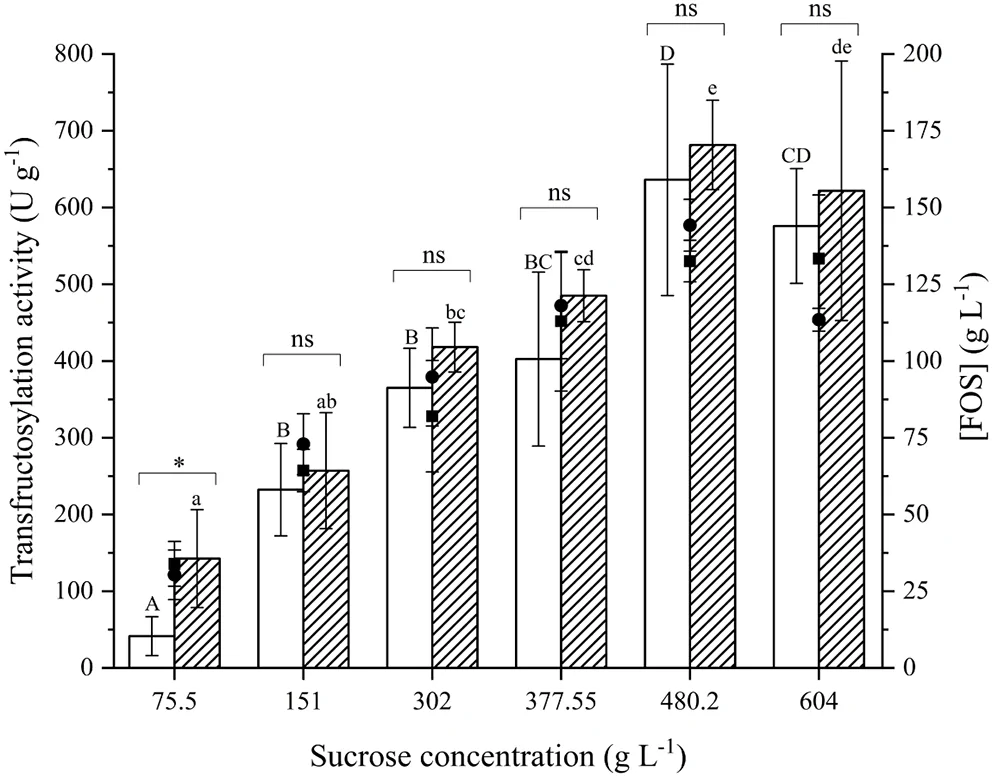
Influence of substrate concentration on the transfructosylation activity of immobilized *A. oryzae* IPT-301 cells in cubic PS matrices (0.8 × 0.8 × 0.8 cm) and on FOS concentration. The enzymatic reaction was performed in a reaction medium consisting of a sucrose solution in 0.2 mol L^–1^ Tris-acetate buffer (pH 5.5), at 190 rpm and 50 °C for 60 min. Transfructosylation activity of IC (open bars) and CIC (hatched bars). FOS concentration for IC (●) and CIC (■). Data are expressed as mean ± SD (*n* = 3). Error bars represent the standard deviation. Different uppercase and lowercase letters indicate statistically significant differences among sucrose concentrations for IC and CIC, respectively, according to Tukey’s HSD test (*p* < 0.05). For comparisons between IC and CIC at the same sucrose concentration, ns indicates no statistically significant difference, whereas * indicates a statistically significant difference (*p* < 0.05).

In previous studies, Cunha et al.,^5^ Garcia et al.,^20^ and Gonçalves et al.^19^ also reported maximum values of transfructosylation activities at similar sucrose concentrations for *A. oryzae* IPT-301 cells *in natura*, cross-linked with glutaraldehyde, and entrapped in calcium alginate, respectively. This indicates that the immobilization of *A. oryzae* IPT-301 cells on the PS matrix, as well as the further cross-linking step, did not alter the behavior of its transfructosylation activity as a function of sucrose concentration. The absolute activity values obtained in this experiment were higher than those observed during the cultivation-time assay (Figure 2), although the same activity definition and normalization basis were used. This difference may be associated with batch-to-batch variability inherent to the *in situ* colonization of a macroporous support by fungal cells. Even under standardized cultivation conditions, variations in the extent and spatial distribution of cell adhesion and growth within the PS may affect the amount and accessibility of catalytically active cells and, consequently, the transfructosylation activity.^8,18^

It was also observed that the highest FOS concentrations, reaching 144.2 g L^–1^ and 132.5 g L^–1^, were obtained at a sucrose concentration of 480.2 g L^–1^ for the IC and CIC on PS, respectively, which is consistent with the maximum transfructosylation activity. Kawee-Ai et al.^43^ evaluated the use of the commercial enzymatic preparation Pectinex Ultra SP-L for FOS synthesis and reported a maximum FOS concentration of 346.78 g L^–1^ at a sucrose concentration of 600 g L^–1^ in a batch system operated at 30 °C and 300 MPa for 15 min. The lowest FOS concentration (approximately 32.1 g L^–1^) was obtained when the lowest sucrose concentration was used. A similar behavior was reported by Sánchez et al.,^44^ who quantified FOS production over a wide range of initial sucrose concentrations (300 g L^–1^ to 800 g L^–1^) in a batch reactor operated at 60 °C and pH 5.5 for 320 min using a β-fructofuranosidase from *Aspergillus* sp. N74, and obtained the minimum FOS concentration (96.98 g L^–1^) at an initial sucrose concentration of 300 g L^–1^. These results indicate that the biocatalysts synthesized in this work have great potential for enzymatic FOS production. Figure 6 shows the fitting of the Michaelis–Menten and zero-order kinetic models to the experimental reaction rates obtained for IC and CIC.

**Figure 6:**
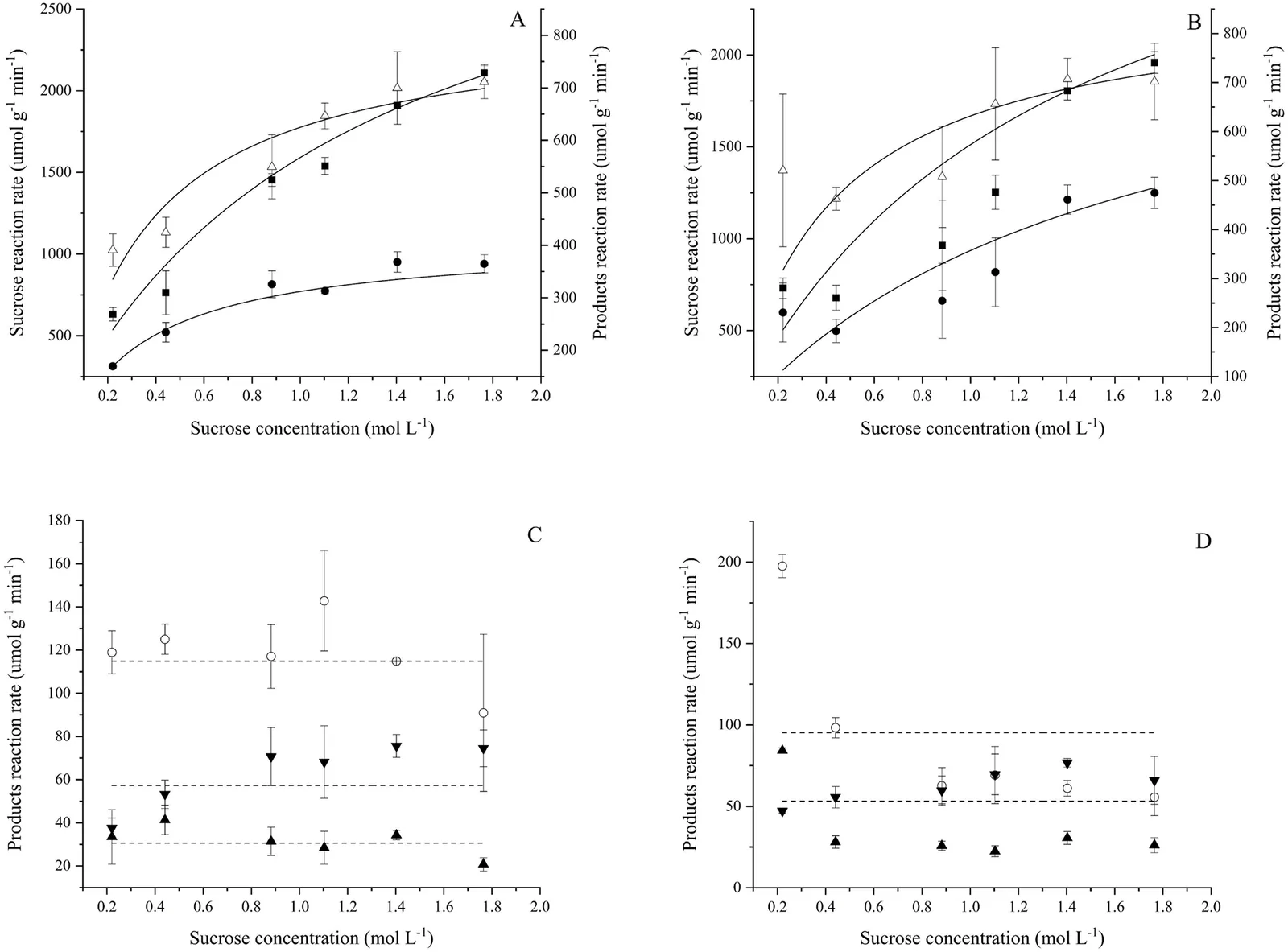
Fitting of the Michaelis–Menten kinetic model to the concentrations of sucrose (■), 1-kestose (●), and glucose (△) for IC (A) and CIC (B) in cubic PS matrices (0.8 × 0.8 × 0.8 cm). Fitting of the zero-order kinetic model to the concentrations of nystose (▼), 1-*β*-fructofuranosylnystose (▲), and fructose (○) for IC (C) and CIC (D). Enzymatic reactions were performed in a reaction medium containing sucrose solution in 0.2 mol L^–1^ Tris-acetate buffer (pH 5.5) at 50 °C and 190 rpm for 60 min. The continuous lines represent the Michaelis–Menten kinetic model, whereas the dashed lines represent the zero-order kinetic model.^23,25^ Data are expressed as mean ± SD (*n* = 3). Error bars represent the standard deviation.

The maximum reaction rate (*V*_max_) of sucrose for IC and CIC was approximately 3500 *μ*mol g^–1^ min^–1^ for both biocatalysts (Table 1). This parameter represents the maximum reaction rate asymptotically reached when the enzyme is fully saturated with the substrate.^25^ Also, low values of the Michaelis–Menten dissociation constant (K_m_), as those obtained in this work, indicate a great enzyme affinity for the substrate molecule.^25^ It can be observed that both biocatalysts exhibited a greater selectivity for sucrose conversion into 1-kestose and glucose, which is characteristic of the transfructosylation reaction mechanism, wherein sucrose donates a fructosyl group to another sucrose molecule, obtaining glucose as a byproduct.^47–49^ Sánchez et al.^44^ reported a $V_{máx}^{app}$ of approximately 200847 *μ*mol L^–1^ h^–1^ for sucrose, as well as 24859 *μ*mol L^–1^ h^–1^ and 2565 *μ*mol L^–1^ h^–1^ for 1-kestose and nystose, respectively.

**Table 1:** Kinetic Parameters and Coefficient of Determination for Immobilized Cells (IC) and Crosslinked Immobilized Cells (CIC) in Cubic PS Matrices (0.8 × 0.8 × 0.8 cm)^a^

Biocatalyst	Component	Michaelis Menten		Zero-order kineticsk (*μ*mol g^–1^ min^–1^)	R^2^
IC	**[S]**	V_max_ (*μ*mol g^–1^ min^–1^)	3582.89 ± 472.99		0.98
		K_m_ (mol L^–1^)	1.25 ± 0.34		
	**GF**_**2**_	V_max_ (*μ*mol g^–1^ min^–1^)	409.43 ± 12.29		0.99
		K_m_ (mol L^–1^)	0.31 ± 0.02		
	**GF**_**3**_			57.26 ± 7.48	
	**GF**_**4**_			30.59 ± 2.84	
	**G**	V_max_ (*μ*mol g^–1^ min^–1^)	826.88 ± 64.00		0.91
		K_m_ (mol L^–1^)	0.32 ± 0.09		
	**F**			114.85 ± 0.13	
CIC	**[S]**	V_max_ (*μ*mol g^–1^ min^–1^)	3481.62 ± 1026.10		0.94
		K_m_ (mol L^–1^)	1.30 ± 0.79		
	**GF**_**2**_	V_max_ (*μ*mol g^–1^ min^–1^)	913.06 ± 269.63		0.91
		K_m_ (mol L^–1^)	1.56 ± 0.83		
	**GF**_**3**_			53.26 ± 5.15	
	**GF**_**4**_			52.87 ± 12.94	
	**G**	V_max_ (*μ*mol g^–1^ min^–1^)	878.30 ± 76.00		0.91
		K_m_ (mol L^–1^)	0.39 ± 0.09		
	**F**			95.20 ± 22.85	

a Sucrose ([S]), Glucose (G), Fructose (F), 1-Kestose (GF_2_), Nystose (GF_3_) and 1-*β*-Fructofuranosylnystose (GF4).

The production rates of nystose, 1-β-fructofuranosylnystose, and fructose were low and showed only slight variation with increasing initial sucrose concentration, allowing a satisfactory fit to the zero-order kinetic model (Table 1). For nystose and 1-β-fructofuranosylnystose, this behavior is associated with an increase in the oligosaccharide’s degree of polymerization, which increases steric hindrance between the molecule and the enzyme’s active site, thereby reducing its production rate.^43–48^ In the case of fructose, slower production rates may be associated with its incorporation into the oligosaccharide chain, resulting in a low release of fructosyl groups into the reaction medium.^49,50^

Table 2 shows that the conversion values for IC and CIC were higher at concentrations below 151 g L^–1^, reaching 75.58% and 68.32%, respectively. However, the highest FOS production was obtained at a concentration of 480.2 g L^–1^ (Figure 5), which resulted in conversion values of 55.56% for IC and 56.93% for CIC.

**Table 2:** Selectivity and Conversion of Immobilized Cells (IC) and Crosslinked Immobilized Cells (CIC) in Cubic PS Matrices (0.8 × 0.8 × 0.8 cm)[Table-fn tbl2fn1],[Table-fn tbl2fn2]

			Selectivity (%)				
Biocatalyst	[S]_0_ (g L^–1^)	X (%)	GF_2_	GF_3_	GF_4_	G	F
IC	75.5	75.58 ± 4.02^A^	22.61 ± 0.22^A^	5.01 ± 0.50^A^	4.46 ± 1.37^AB^	52.07 ± 2.7^A^	15.85 ± 0.94^A^
	151.0	57.73 ± 8.24^AB^	26.67 ± 1.75^AB^	6.06 ± 0.61^A^	4.70 ± 0.63^B^	48.33 ± 2.31^A^	14.23 ± 0.56^A^
	302.0	57.19 ± 7.91^AB^	29.77 ± 1.91^B^	6.45 ± 1.00^A^	2.87 ± 0.49^ABC^	50.21 ± 3.94^A^	10.70 ± 0.95^AB^
	377.6	61.23 ± 3.95^AB^	26.13 ± 0.26^AB^	5.68 ± 1.14^A^	2.37 ± 0.52^AC^	53.91 ± 1.44^A^	11.91 ± 1.37^AB^
	480.2	50.55 ± 8.74^B^	28.49 ± 1.23^B^	5.85 ± 0.33^A^	2.65 ± 0.13^ABC^	54.12 ± 4.37^A^	8.88 ± 0.01^AB^
	604.0	51.41 ± 1.03^B^	28.90 ± 1.12^B^	5.90 ± 0.55^A^	1.65 ± 0.20^C^	56.34 ± 1.76^A^	7.20 ± 2.36^B^
CIC	75.5	58.25 ± 9.48^a^	21.35 ± 3.94^a^	4.36 ± 0.09^a^	7.80 ± 0.11^a^	48.02 ± 10.20^a^	18.29 ± 0.46^a^
	151.0	63.80 ± 4.52^a^	23.03 ± 2.32^a^	6.63 ± 0.55^a^	3.35 ± 0.37^b^	55.24 ± 1.98^a^	11.73 ± 0.52^b^
	302.0	41.92 ± 11.85^a^	27.97 ± 6.86^a^	6.55 ± 0.70^a^	2.83 ± 0.25^bc^	55.76 ± 8.0^a^	6.88 ± 0.86^c^
	377.6	56.13 ± 3.23^a^	27.69 ± 4.37^a^	6.15 ± 0.78^a^	1.98 ± 0.21^c^	58.04 ± 7.16^a^	6.12 ± 1.10^c^
	480.2	56.93 ± 2.44^a^	34.50 ± 1.82^a^	5.74 ± 0.16^a^	2.28 ± 0.24^c^	52.90 ± 2.59^a^	4.56 ± 0.26^c^
	604.0	54.50 ± 2.18^a^	35.84 ± 1.70^a^	4.98 ± 0.78^a^	1.97 ± 0.28^c^	53.00 ± 4.16^a^	4.19 ± 0.70^c^

i Note: Values are expressed as mean ± standard deviation (SD) of three independent measurements (*n* = 3). For each parameter (X and selectivity), statistical comparisons among initial sucrose concentrations were performed separately for IC and CIC using ANOVA followed by Tukey’s HSD test (*p* < 0.05). Different uppercase and lowercase letters within each parameter indicate statistically significant differences among initial sucrose concentrations for IC and CIC, respectively.

ii Initial Sucrose Concentration ([S]_0_), Conversion (X), Glucose (G), Fructose (F), 1-Kestose (GF_2_), Nystose (GF_3_) and 1-*β*-Fructofuranosylnystose (GF4).

Both evaluated biocatalysts exhibited great conversion selectivity toward 1-kestose and glucose (Table 2). The average values of 1-kestose selectivity for IC and CIC were 27.09% and 28.23%, respectively, which were 4.6 and 4.9 times higher than those obtained for nystose. This is an important result, since the lower the degree of polymerization of FOS, the greater their nutraceutical properties.^48^

Despite the favorable production of 1-kestose, the selectivity of the present biocatalytic system was lower than that reported for other FOS-producing systems. Sánchez et al.^44^ reported a selectivity of up to 93.6% toward 1-kestose and nystose using β-fructofuranosidase from *Aspergillus* sp. N74 at a sucrose concentration of 550 g L^–1^, while no production of 1-β-fructofuranosylnystose was detected. Likewise, studies evaluating the production rate and selectivity of the enzymatic preparation Pectinex Ultra SP-L reported predominant production of 1-kestose, with low production of nystose and 1-β-fructofuranosylnystose.^43,49^ On the other hand, the high glucose selectivity, which reached approximately 52.5% and 53.8% (Table 2) for IC and CIC, respectively, can be associated with the reaction mechanism, wherein glucose is a byproduct of both sucrose hydrolysis and transfructosylation.^48,49,51,52^ Although glucose formation is therefore an inherent consequence of the reaction pathways involved in sucrose conversion, its high proportion may limit both FOS yield and product purity.^53,54^ These results indicate that, despite the operational advantages provided by the proposed immobilization strategy, further optimization is required to favor sucrose transfructosylation over competing hydrolytic reactions.^5,9^ Therefore, increasing the fraction of sucrose converted into FOS while simultaneously reducing glucose formation represents an important step toward improving the performance of this biocatalytic system.

### Operational Stability

Figure 7 shows the operational stability of the biocatalysts over 12 consecutive reaction cycles in batch mode. It was observed that, at the end of 12 reaction cycles, the IC and CIC biocatalysts retained 50.23 ± 11.90% and 60.90 ± 5.30% of their relative transfructosylation activities, respectively. The maximum transfructosylation activities of the IC and CIC were 922.77 ± 85.56 U g^–1^ and 1089.95 ± 99.48 U g^–1^, respectively, and these values were defined as 100% for the calculation of the relative activity. Garcia et al.,^20^ who reported an increase in operational stability by cross-linking *A. oryzae* IPT-301 cells with glutaraldehyde, observed only 11.1% of the initial activity after 12 cycles. In turn, the operational stability studies conducted by Gonçalves et al.^19^ showed that the transfructosylation activity of *A. oryzae* IPT-301 cells cross-linked and encapsulated in calcium alginate retained up to 52% of the initial activity after 12 reaction cycles.

**Figure 7:**
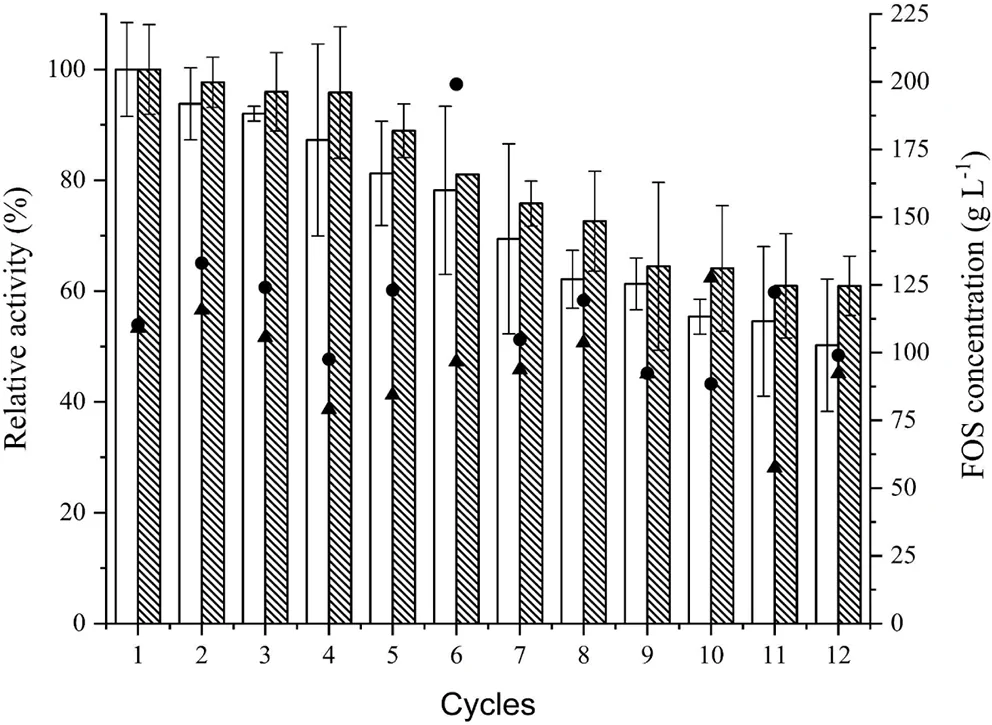
Operational stability of IC and CIC in cubic PS matrices (0.8 × 0.8 × 0.8 cm) and FOS concentration during consecutive batch reaction cycles. Transfructosylation activity of IC (open bars) and CIC (hatched bars); FOS concentration for IC (▲) and CIC (●). Reaction conditions: 48% (w v^–1^) sucrose solution, 0.2 mol L^–1^ Tris-acetate buffer (pH 5.5), 190 rpm, 50 °C, and 60 min. Data are expressed as mean ± SD (*n* = 3). Error bars represent the standard deviation.

Complementing the operational stability data presented in Figure 7, Table 3 summarizes the performance parameters (conversion, yield, and productivity) calculated from eqs 5–7 for the batch-operated system. Both biocatalysts (IC and CIC) exhibited high operational stability over 12 consecutive reaction cycles, with only a minor loss of activity (Figure 7). The IC achieved sucrose-to-FOS conversions ranging from 25.6% to 41.7%, whereas the CIC showed values ranging from 30.2% to 42.8%. The yield ranged from 20.5% to 28.3% for IC and from 20.1% to 29.4% for CIC, with productivity exceeding 96 g L^–1^ h^–1^ for both biocatalysts. Based on these descriptive results, the cross-linking process did not appear to improve the performance parameters or the biochemical properties of *A. oryzae* IPT-301 cells immobilized on PS. The IC exhibited average conversion and yield values for FOS production of 35.2% and 23.6%, respectively, with an average productivity of 113.2 g L^–1^ h^–1^. Similarly, the CIC achieved average conversion, yield, and productivity values of 36.3%, 24.7%, and 118.6 g L^–1^ h^–1^, respectively. Although previous studies have shown that the cross-linking process significantly increased the operational stability of *A. oryzae* IPT-301 cells that were not immobilized on porous support materials during microbial cultivation,^20^ the results of this study demonstrated that cell cross-linking after *in situ* immobilization on PS did not improve the performance of the biocatalyst under the conditions evaluated. The macroporous structure of PS was sufficient to provide stability and enable the reuse of the immobilized cells. Thus, under the conditions evaluated, the additional cross-linking step was not necessary. Eliminating this step may simplify biocatalyst preparation, reduce the use of chemical reagents, and decrease the number of processing steps.

**Table 3:** Performance Parameters (Conversion, Yield, and Productivity) Obtained for Immobilized Cells (IC) and Crosslinked Immobilized Cells (CIC) of *A. oryzae* IPT-301

Batch operation cycle	Biocatalyst	Conversion (X)(%)	Yield (Y)(%)	Productivity (P)(g L^–1^ h^–1^)
1°	IC	40.9	24.5	117.9
	CIC	30.2	28.1	134.8
2°	IC	33.5	25.8	123.8
	CIC	38.9	29.4	141.0
4°	IC	31.6	20.5	98.6
	CIC	32.5	23.5	113.0
6°	IC	41.7	21.8	104.8
	CIC	35.3	23.2	111.5
8°	IC	37.7	23.3	111.9
	CIC	42.8	26.5	127.2
10°	IC	35.6	28.3	135.8
	CIC	31.8	20.1	96.7
12°	IC	25.6	20.8	99.6
	CIC	42.5	22.1	106.0

Several studies have focused on optimizing the operational parameters for FOS production using both whole and immobilized cells. Khatun et al.^55^ reported FOS yield and productivity values of 61.2% and 102 g L^–1^ h^–1^, respectively, employing lyophilized cells of *Aureobasidium pullulans* FRR 5284. In addition, Choukade and Kango^56^ achieved an FOS concentration of 325 g L^–1^ with a yield of 55% using whole cells of *Aspergillus tamarii* NKRC 1229. Castro et al.^8^ evaluated the immobilization of *Aureobasidium pullulans* cells on reticulated polyurethane foam, obtaining increases in FOS concentration, purity, and yield of 15%, 8%, and 12% (w w^–1^), respectively, compared with free-cell conditions. In turn, Huang et al.^16^ obtained a maximum FOS yield of 65.47% (w w^–1^) employing *Aspergillus aculeatus* M105 cells immobilized in calcium alginate. Mussatto et al.^57^ evaluated FOS production by *Penicillium expansum* cells immobilized on synthetic fiber and polyurethane foam, resulting in FOS concentrations of 120.3 g L^–1^ and 104.8 g L^–1^, respectively. Finally, Jung et al.^58^ investigated continuous FOS production in a pilot-scale packed bed reactor using *Aureobasidium pullulans* cells immobilized in calcium alginate beads, achieving a productivity of 180 g L^–1^ h^–1^. These differences in FOS yield and productivity should be interpreted considering the different microorganisms, biocatalyst preparation methods, immobilization strategies, substrate concentrations, reaction times, and reactor configurations employed in these studies. In the present work, the lower FOS yield may be associated with the high glucose formation observed during sucrose conversion (Table 2). Although glucose is inherently released during transfructosylation, it can also be formed through sucrose hydrolysis. Thus, the high selectivity toward glucose, together with the lower selectivity toward FOS (GF_2_, GF_3_ and GF_4_), may indicate the concurrent occurrence of sucrose hydrolysis and transfructosylation, which may have contributed to the lower FOS yield observed in the present study. In contrast, the average productivities obtained for IC and CIC (113.2 and 118.6 g L^–1^ h^–1^, respectively) indicate substantial FOS formation within the short reaction time employed (1 h batch cycle). Despite the lower FOS yield, the proposed approach combines competitive productivity with direct *in situ* immobilization of *A. oryzae* IPT-301 cells on a macroporous support, enabling biocatalyst reuse without an additional cross-linking step.

The present study was conducted in batch mode at laboratory scale, and therefore the behavior of the immobilized biocatalyst under continuous operation, particularly in packed-bed reactors, remains to be demonstrated. This represents an important step toward process scale-up, since recent studies have highlighted reactor configuration, enzyme stability, and product yield as key factors in the development of efficient large-scale FOS production processes.^59^ Parameters such as pressure drop, mechanical stability of the sponge, long-term cell retention, mass-transfer effects at higher bed heights, and performance under prolonged continuous operation should be evaluated.^12,22,60^ Thus, although the high operational stability observed over 12 consecutive cycles indicates promising reusability, further studies are required to establish the suitability of the PS-based biocatalyst for continuous and large-scale FOS production.

### Thermal Stability and Kinetic and Thermodynamic Parameters

The thermostability profiles of both IC and CIC biocatalysts are presented in Figure 8A and Figure 8B, respectively. After incubation at 30 °C for 36 h, the IC biocatalyst retained 58.2 ± 9% of its initial activity (272.6 U g^–1^), whereas the CIC biocatalyst maintained a higher residual activity of 84.8 ± 4% (448.8 U g^–1^). At the end of the incubation period, the CIC biocatalyst exhibited higher thermal stability (68.8 ± 11%, equivalent to 364.13 U g^–1^) than the IC biocatalyst (57.8 ± 2%, equivalent to 270.93 U g^–1^) at 40 °C. The results suggest that the cross-linking of immobilized cells with glutaraldehyde makes the biocatalyst thermally and chemically stable, as it reacts rapidly with the protein’s amine groups to form intra- and intermolecular covalent bonds, which makes the biocatalyst more rigid and, therefore, resistant to conformational changes, giving it greater resistance to denaturation by heat.^61–63^ On the other hand, the residual activity values decreased with increasing temperature (50 and 60 °C) and incubation time, indicating the irreversible thermal denaturation of the biocatalysts (Figure 8). Previous research with *A. oryzae* IPT-301 whole cells^5^ and encapsulated cells in calcium alginate^19^ showed greater thermal stability for a 30 to 40 °C range, which corroborates the results of this work.

**Figure 8:**
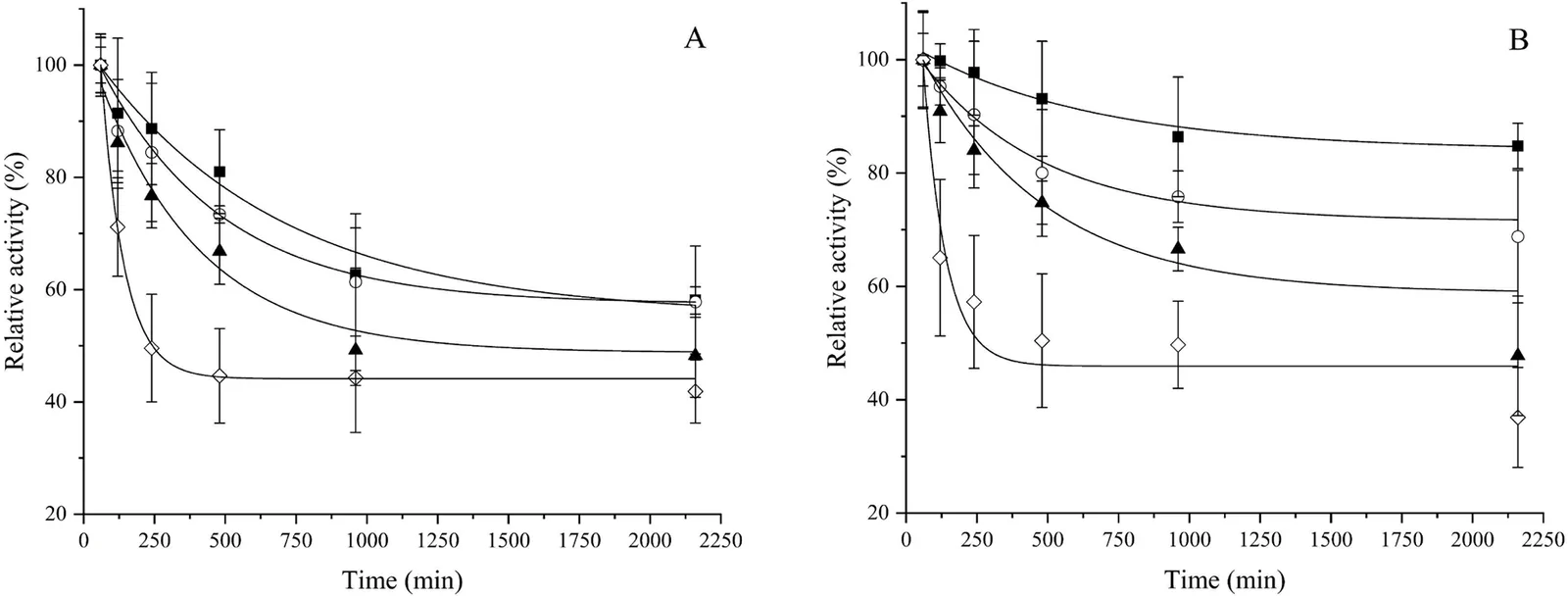
Thermostability profiles of immobilized cells—IC (A) and cross-linked immobilized cells—CIC (B) on polyurethane sponge over 36 h of incubation at different temperatures: 30 °C (■), 40 °C (○), 50 °C (▲) and 60 °C (◊). Continuous line: Sadana and Henley (1987) thermal inactivation model fitted to the experimental data. The maximum activity of the IC (468.74 ± 22.29 U g^–1^) and CIC (529.26 ± 44.21 U g^–1^) biocatalysts was defined as 100% relative activity. Data are expressed as mean ± SD (*n* = 3). Error bars represent the standard deviation.

Table 4 presents the inactivation kinetic constants (k_D_, *t*_1/2_, and E_D_) and thermodynamics parameters (ΔH_D_; ΔS_D_ and ΔG_D_) for both IC and CIC biocatalysts. These data provide insight into the resistance of the biocatalysts to the unfolding process.^64^

**Table 4:** Inactivation Kinetics Constants and Thermodynamic Parameters Obtained for the *A. oryzae* IPT-301 Biocatalytic Immobilized Cells (IC) and Crosslinked Immobilized Cells (CIC) on a Polyurethane Sponge (PS)^a^

		Temperature (°C)			
Parameter	Biocatalyst	30	40	50	60
R^2^	IC	0.98	0.99	0.97	0.99
	CIC	0.99	0.99	0.98	0.94
k_D_ (min^–1^)	IC	1.52 × 10^–3^	2.32 × 10^–3^	2.78 × 10^–3^	12.33 × 10^–3^
	CIC	1.59 × 10^–3^	2.32 × 10^–3^	2.28 × 10^–3^	12.69 × 10^–3^
t_1/2_ (min)	IC	456.01	298.78	249.33	56.21
	CIC	435.94	298.77	304.01	54.62
E_D_ (kJ mol^–1^)	IC	53.94			
	CIC	51.39			
ΔH_D_ (kJ mol^–1^)	IC	51.42	51.34	51.25	51.17
	CIC	48.87	48.79	48.71	48.63
ΔG_D_ (kJ mol^–1^)	IC	100.91	103.22	106.12	105.37
	CIC	100.79	103.22	106.65	105.28
ΔS_D_ (kJ mol^–1^ K^–1^)	IC	–0.1633	–0.1657	–0.1698	–0.1627
	CIC	–0.1713	–0.1739	–0.1793	–0.1701

a R^2^: correlation coefficient for the *k*_D_ values; *k*_D_: first-order thermal denaturation constant; *t*_1/2_: half-life of the biocatalyst; *E*_D_: activation energy of denaturation; Δ*H*_D_: enthalpy of activation of denaturation; Δ*G*_D_: Gibbs energy of activation of denaturation; Δ*S*_D_: entropy of activation of denaturation.

Inactivation kinetic constants for both IC and CIC biocatalysts indicated a decline in transfructosylation activity over incubation time. The first-order thermal denaturation constant (k_D_) increased, and half-life (*t*_1/2_) decreased progressively with increasing temperature, indicating thermal inactivation of the biocatalyst. The *t*_1/2_ values were very similar for both biocatalysts, indicating that cross-linked immobilized cells did not significantly affect enzyme thermostability. Since half-life is the time required for the enzymatic activity to decrease to 50% of its initial value at a given temperature,^21,65,66^ the biocatalysts showed higher reaction rates over a long period, suggesting potential for their application in industrial processes. In addition, the *t*_1/2_ for both IC and CIC biocatalysts at 30 °C were about 1.5- and 8-fold higher than those at 40 and 60 °C, respectively, clearly indicating heat-induced denaturation of the biocatalysts.^67–69^ At 50 °C, the *t*_1/2_ for both IC and CIC biocatalysts was 1.8 and 1.4-fold lower than at 30 °C, respectively.

Table 4 also shows the activation energy of denaturation (E_D_) for both IC and CIC biocatalysts. This kinetic parameter quantifies the energy required to promote the biocatalytic denaturation of the active sites.^24,67–70^ Hence, the higher the E_D_ value, the greater the energy required to denature the protein structure and, therefore, the greater the thermostability of the biocatalyst.^21,65,67^ The E_D_ value of IC (53.94 kJ mol^–1^) was slightly higher than that of CIC (51.39 kJ mol^–1^), suggesting that the cross-linking of the *A. oryzae* IPT-301 biocatalytic cells with glutaraldehyde after immobilization on polyurethane sponge did not affect the thermostability of the biocatalyst. On the other hand, previous studies have reported that biocatalytic cells from the same microorganism cross-linked with glutaraldehyde and encapsulated in calcium alginate showed E_D_ values 4.5-fold higher (136.32 kJ mol^–1^) than those of the *in-nature* cells (30.43 kJ mol^–1^).^19^

Thermal denaturation of biocatalytic cells is always an endothermic process because energy must be supplied to break intramolecular electrostatic interactions, such as hydrogen bonds and hydrophobic interactions; consequently, the enthalpy of activation of denaturation (ΔH_D_) is always positive.^66^ Therefore, ΔH_D_ is an important thermodynamic parameter associated with the total amount of energy necessary to denature the biocatalytic cells.^71,72^ High and positive values of this parameter indicate high thermostability of the biocatalyst.^67,69,72^ The biocatalytic cells investigated here showed high, positive ΔH_D_ values across the entire temperature range evaluated (Table 4), indicating that both biocatalysts exhibited thermostable behavior.

Table 4 also shows the entropy of activation of denaturation (ΔS_D_) and Gibbs energy of activation of denaturation (ΔG_D_) values. ΔS_D_ expresses the amount of energy per degree of disorder involved in the transition from the native to a denatured state.^19,21,24^ Negative ΔS_D_ values were obtained for both biocatalysts at all incubated temperatures evaluated, indicating a transition to a more ordered state.^71,72^ It is worth noting that increasing temperature can weaken polar enzyme interactions while strengthening hydrophobic interactions. This may explain the negative ΔS_D_ values are obtained because the enzyme’s resistance to unfolding, driven by stronger hydrophobic interactions, outweighs the tendency for the enzyme structure to be disrupted by weakened polar interactions at high temperatures.^65,68,69,73^ In addition, ΔS_D_ can be reduced by water molecules forming cage-like ordered structures around the partially exposed hydrophobic residues of the enzyme, making the unfolded intermediate state more ordered than the native state.^66^

In turn, ΔG_D_ values arise from the stabilizing forces in the molecular structure of enzymes, including hydrogen bonds and *van der Waals* interactions.^67^ Also, this parameter considers the contributions of both enthalpy and entropy to the process spontaneity and, therefore, it is the most reliable and precise parameter to evaluate the biocatalyst’s thermal stability.^65,69,72^ Table 4 shows that positive ΔG_D_ values were obtained for both biocatalysts, indicating their high thermostability.^73,74^ Positive ΔG_D_ values also suggest that, under equilibrium conditions, the concentration of the protein in the native state is higher than in the denatured state.^65,67^ This suggests that for both biocatalysts, the transition of the FTase from the native to the denatured state is a nonspontaneous thermal denaturation process.^19,67^

### pH Incubation Stability and Storage Stability

After 24 h of storage at different pH values, both IC and CIC biocatalysts maintained high transfructosylation activity over a broad pH range (Figure 9). The highest mean transfructosylation activities for both biocatalysts were observed within the storage pH range of 4.5–6.0. The highest mean activity values were observed at pH 5.5, reaching 536.86 ± 4.74 and 605.88 ± 37.62 U g^–1^ for IC and CIC, respectively. Although CIC showed numerically higher mean activity than IC at most of the evaluated pH values, no statistically significant differences were observed between the two biocatalysts at the same storage pH (*p* > 0.05). In addition, for both IC and CIC, the activity at pH 3.5 was significantly lower than that observed at the other evaluated pH values (*p* < 0.05). Gonçalves et al.^19^ reported that the same microorganism was stable over the pH range of 4.5–6.5 for free cells and 5.5–7.5 when encapsulated in calcium alginate beads.

**Figure 9:**
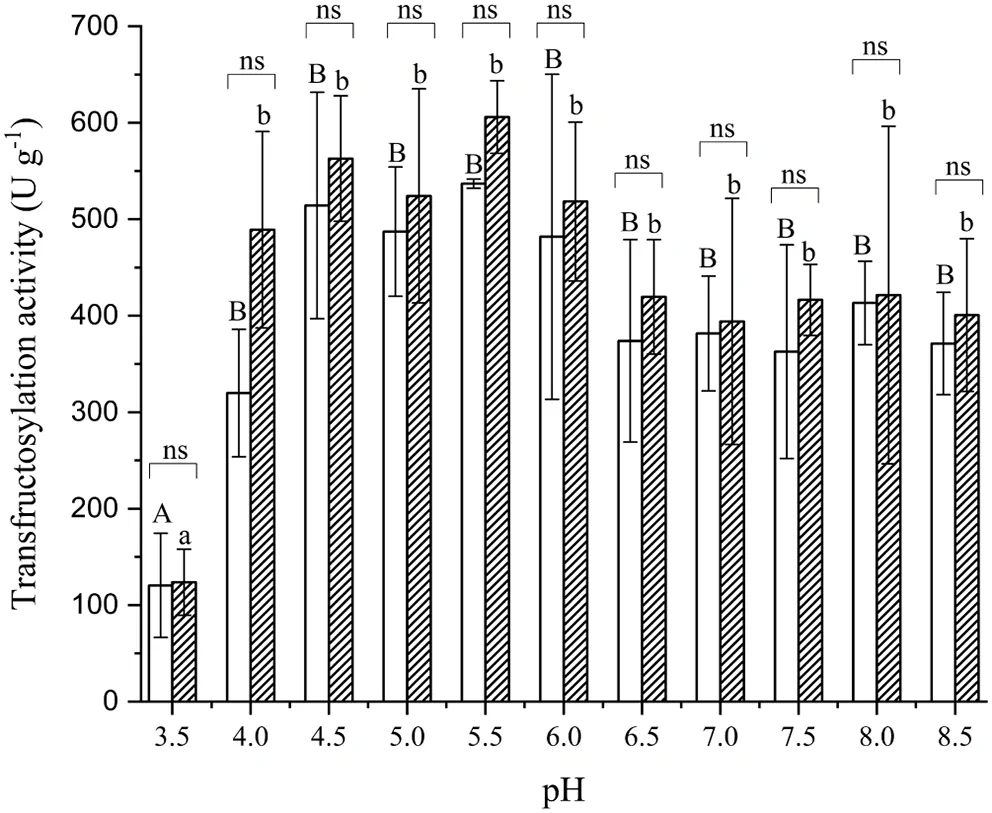
pH stability profile of IC and CIC in cubic PS matrices (0.8 × 0.8 × 0.8 cm). Transfructosylation activity of IC (open bars) and CIC (hatched bars). Reaction conditions: 48% (w v^–1^) sucrose solution, 0.2 mol L^–1^ Tris-acetate buffer, 190 rpm, 50 °C, and 60 min. Data are expressed as mean ± SD (*n* = 3). Error bars represent the standard deviation. Different uppercase and lowercase letters indicate statistically significant differences in pH for IC and CIC, respectively, according to Tukey’s HSD test (*p* < 0.05). For comparisons between IC and CIC at the same pH, ns indicates no statistically significant difference, whereas * indicates a statistically significant difference (*p* < 0.05).

Figure 10 shows the storage stability of the IC and CIC biocatalysts on PS over 28 days of storage. From day 7 to day 21, both biocatalysts retained approximately 40–60% of their initial activity, and at day 28, IC and CIC still retained approximately 44% and 38%, respectively. No statistically significant differences were observed between IC and CIC at the same storage time, except on day 5, when CIC exhibited significantly higher relative activity than IC (*p* < 0.05). These results indicate an improvement in the storage stability of *A. oryzae* IPT-301 cells, which showed only 5 days of stability in the nonimmobilized state.^19^ In a previous study, glutaraldehyde-cross-linked *A. oryzae* IPT-301 cells entrapped in calcium alginate beads retained 95% of their transfructosylation activity after 9 days at 4 °C.^19^ It is worth mentioning that although cell entrapment in alginate gel allows higher storage stability, it also creates a barrier between the substrate and the active sites, thereby decreasing the substrate diffusion rate. In this sense, cell immobilization on the porous PS used in this work resulted in substantial retention of transfructosylation activity during storage (Figure 10). These results highlight the potential of the biocatalysts prepared in this work for future applications in FOS production.

**Figure 10:**
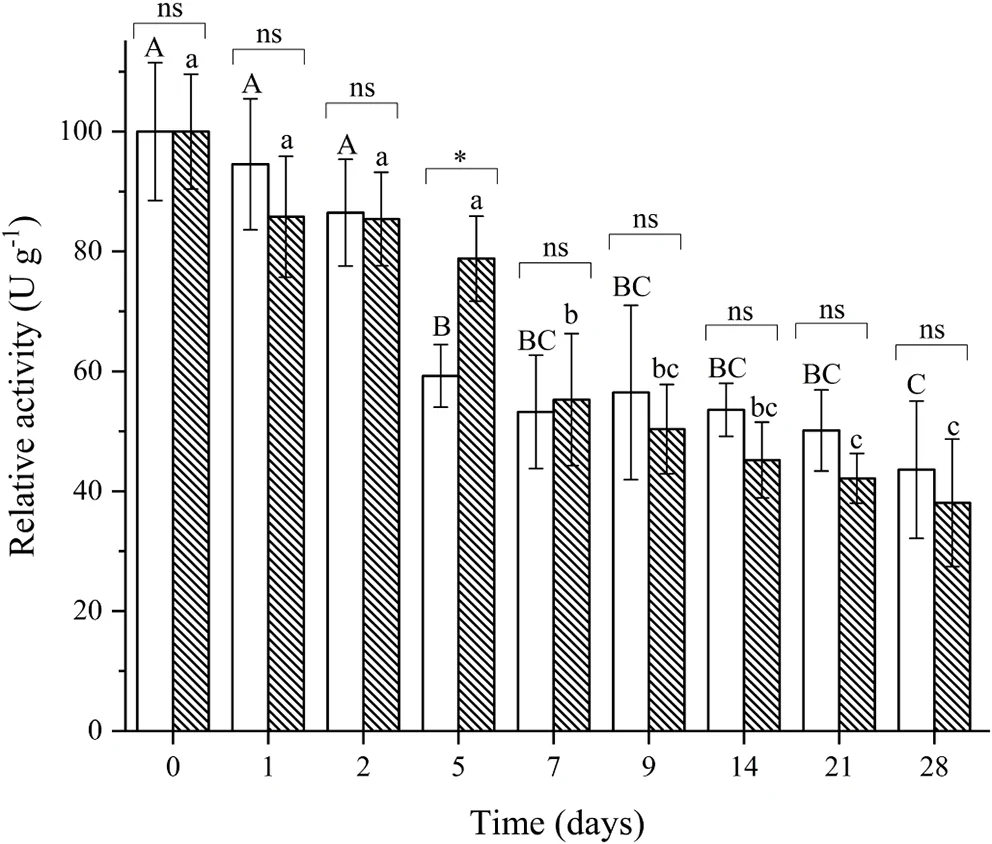
Storage stability profiles of IC and CIC in cubic PS matrices (0.8 × 0.8 × 0.8 cm). Transfructosylation activity of IC (open bars) and CIC (hatched bars). Reaction conditions: 48% (w v^–1^) sucrose solution, 0.2 mol L^–1^ Tris-acetate buffer (pH 5.5), 190 rpm, 50 °C, and 60 min. The maximum activities of IC (910.29 ± 98.56 U g^–1^) and CIC (1075.73 ± 97.05 U g^–1^) were defined as 100% relative activity. Data are expressed as mean ± SD (*n* = 3). Error bars represent the standard deviation. Different uppercase and lowercase letters indicate statistically significant differences among storage times (days) for IC and CIC, respectively, according to Tukey’s HSD test (*p* < 0.05). For comparisons between IC and CIC on the same day, ns indicates no statistically significant difference, whereas * indicates a statistically significant difference (*p* < 0.05).

A highly active and stable biocatalyst for FOS production was obtained by immobilizing *A. oryzae* IPT-301 cells on PS during submerged cultivation for 32 h. Furthermore, it was demonstrated that adding a cross-linking step to the immobilization process did not significantly affect the catalytic behavior or stability of cells previously immobilized on PS, indicating that cross-linking is not required under the conditions evaluated. This finding further supports *in situ* immobilization on PS as a simpler biocatalyst preparation strategy that avoids additional chemical treatment. The immobilized cells showed high reuse capacity, as well as high pH, thermal, and storage stability, which are desirable characteristics for industrial biocatalysts. The biocatalyst also enabled the production of high FOS concentrations during 12 consecutive 1 h batch reaction cycles. Therefore, these results demonstrate that this approach provides a simple and rapid strategy for preparing stable biocatalysts for FOS production. The proposed in situ immobilization on polyurethane sponge can also represent a low-cost route for preparing stable biocatalysts for FOS production, with competitive productivity and considerable reusability. Nevertheless, further studies are still required to demonstrate scale-up, particularly in continuous packed-bed reactors, with respect to the catalyst deactivation rate and its mechanical strength under continuous flow. In addition, the high glucose formation observed warrants additional investigation aimed at improving FOS purification.

## Data Availability

The data that support the findings of this study are available from the corresponding author upon reasonable request.
